# Artificial-Intelligence-Based Models Coupled with Correspondence Analysis Visualization on ART—Cases from Gombe State, Nigeria: A Comparative Study

**DOI:** 10.3390/life13030715

**Published:** 2023-03-06

**Authors:** Kabiru Bala, Ilker Etikan, A. G. Usman, S. I. Abba

**Affiliations:** 1 Biostatistics Department, Faculty of Medicine, Near East University, 99138 Nicosia, Cyprus; 2 Taraba State Polytechnic Suntai, Jalingo Campus, Howayi 660213, Taraba, Nigeria; 3 Operational Research Centre in Healthcare, Near East University, 99138 Nicosia, Cyprus; 4 Department of Analytical Chemistry, Faculty of Pharmacy, Near East University, 99138 Nicosia, Cyprus; 5 Interdisciplinary Research Centre for Membrane and Water Security, Faculty of Petroleum and Minerals, King Fahd University, Dhahran 31261, Saudi Arabia

**Keywords:** antiretroviral therapy, artificial intelligence, correspondence analysis, protease inhibitors, Gombe state, Nigeria

## Abstract

Antiretroviral therapy (ART) is the common hope for HIV/AIDS-treated patients. Total commitments from individuals and the entire community are the major challenges faced during treatment. This study investigated the progress of ART in the Federal Teaching Hospital in Gombe state, Nigeria by using various records of patients receiving treatment in the ART hospital unit. We combined artificial intelligence (AI)-based models and correspondence analysis (CA) techniques to predict and visualize the progress of ART from the beginning to the end. The AI models employed are artificial neural networks (ANNs), adaptive neuro-fuzzy inference systems (ANFISs) and support-vector machines (SVMs) and a classical linear regression model of multiple linear regression (MLR). According to the outcome of this study, ANFIS in both training and testing outperformed the remaining models given the R^2^ (0.903 and 0.904) and MSE (7.961 and 3.751) values, revealing that any increase in the number of years of taking ART medication will provide HIV/AIDS-treated patients with safer and elongated lives. The contingency results for the CA and the chi-square test did an excellent job of capturing and visualizing the patients on medication, which gave similar results in return, revealing there is a significant association between ART drugs and the age group, while the association between ART drugs and marital status (93.7%) explained a higher percentage of variation compared with the remaining variables.

## 1. Introduction

The complicated retrovirus (lentivirus) family, known as human immunodeficiency virus (HIV), is a virus that usually threatens human life, resulting in patient death when treatment is not adequately followed or commences early as prescribed by experts in the clinical field. The destruction of the immune system is the major concern of the virus. As a result of this virus attack, medical researchers developed the current antiretroviral therapy (ART) drug that will reduce and stop the further replication of the virus. Currently, the ART drug is the one that is capable and reliable in handling both HIV-1 and HIV-2 cases [1].

Several researchers have used different statistical models to predict its epidemiology, such as HIV/AIDS-related cases and their development, which have resulted in the current achievement of undetectable and non-transmissible outcomes among people living with the virus [2,3,4]. Some researchers used logistic regression to predict the influence of the early risk factors of SARS-CoV-2 ART associated with the advancement of the disease, and the findings showed that vaccination and early treatment with antivirals have significantly reduced the risk of disease progression. Similarly, the chi-square test and stepwise regression to investigate the longer duration of ART in overcoming barriers to long-term adherence, improving the survival of adolescents and young adults with HIV, and the results supported the global goals for HIV prevention and treatment. Zalla et al. [3] employed binomial distribution to examine the differences in race and ethnicity among people entering HIV care for ART medication, and the results indicated no significant difference in ART medication according to race or ethnicity, but Black and Hispanic patients showed a significant difference compared with White patients receiving ART. Torres et al. [5] used a chi-square analysis and a *t* test to evaluate the frequency incidence difference between S68G and K65R mutations on the basis of the different types of HIV-1.

Similarly, Kiyingi et al. [6] confirmed that the regression analysis results showed that women living with HIV have a high risk of human papillomavirus infection. However, Hameiri-bowen et al. [7] concluded that there was a significant delay in prevalence and associated factors of ART launching among people living with HIV on the basis of the PCA and logistic regression results. Chaula et al. [8] confirmed that the PCA findings showed there was no sustained medication interruption after examining the effect of azithromycin on the illustration of plasma-soluble biomarkers in adults and children who had chronic lung disease. Jin et al. [9] employed logistic regression and PCA to determine whether the HIV-antibody repertoire can be used to predict the viral reservoir, and the PCA results showed that there was a correlation between the DNA of HIV patients and western blot kits; in addition, the variables were associated with the prevalence, according to the clustered indicators.

Camargo et al. [10] confirmed that the study utilized by MCA demonstrated a relationship between variables related to HIV/AIDS patients’ failure to adhere to ART. Bayon et al. [11] investigated the serum levels of amylase and CD4 in newly diagnosed people living with HIV by using a chi-square test and MLR, and the results suggested that opportunistic infections caused by low CD4 counts could be the primary cause of pancreatic damage. Soogun et al. [12] applied LG and CA to forecast the multidrug support of a staphylococcus aureus nasal isolate with clonal complex in HIV-treated patients, and the CA demonstrated positive associations. Chaula et al. [13] employed correlation and a mixed-effects statistical model to predict the early duration of ART treatment to stabilize the growth of viral reservoirs in HIV-1 patients, and the results showed that early treatment can to some extent decrease the viral load. Alanzi et al. [14] presented factors related to high viral load by using MCA and RF, and the outcomes showed that MCA was the tool that best meets the UNAIDS 95–95–95 target goal in achieving viral load suppression in that the study identified a high proportion of individuals with a lower viral load.

However, several scholars have used AI models to predict the achievement and progress of patients surviving HIV/AIDS. Other such as Rodriguez et al. [15] used RF machine-learning techniques to forecast the history of HIV-treated breastfeeding mothers, and the result showed that RF gave a higher accuracy performance. Besides, artificial neural networks (ANNs) was employed to estimate prokaryotic genomes’ important genes, and the findings revealed that an ANN is the most efficient model. Dimopoulos et al. [16] used the deep-learning method to evaluate empathic conduct in a social dynamic condition, and the results showed that the proposed method surpasses other popular ML methods and did so with high maximum accuracy. Vapnik, et al. [17] developed the conceptual overview of AI based SVM theory and logistic regression, and the results showed that ML techniques such as SVM surpassed logistic regression. In addition, Farhat [18] utilized the radial basis function, SVM, product unit and sigmoid unit to investigate the typology of HIV-treated patients receiving ART medication, and the results showed that radial basis neural networks demonstrated the best performance accuracy.

This study aims to integrate four AI-based models and CA techniques to predict the outcomes of HIV/AIDS patients receiving ART at Federal Teaching Hospital in Gombe state, Nigeria. The proposed AI algorithm was designed to compare the performance of three nonlinear (ANN, ANFIS and SVM) models and one linear (MLR) model. The main purpose of employing AI is to evaluate the influence and effect of taking ART medication for a longer duration while the CA is employed, to examine the visual positioning and contributions of each row and column term as dimensions of the success for the ART medication. The authors have not yet seen any related paper combining artificial intelligence models and correspondence analysis. Section 2 of this paper contains the presentation of material and methods connected to HIV/AIDS examination. This is followed by a discussion of the proposed approach in Section 3 and a conclusion in Section 4.

## 2. Materials and Methods

### 2.1. Study Population

The study population consists of 2500 HIV/AIDS patients taking ART at the hospital; most of the patients come from various states of the geopolitical zone comprising Gombe, Bauchi, Yobe, Borno, Adamawa and Taraba. Sociodemographic variables of interest used in retrieving the variables from the ART database of the hospital include age, gender, marital status, hospital status, state visit, viral load and regimen combination of ART drugs.

### 2.2. Study Design and Analysis Method

A retrospective cohort study was adopted to select the patients visiting the hospital for their ART medication. Patients’ records on ART were randomly selected from the HIV/AIDS hospital database. AI models were employed to predict the ages of patients on follow-up, while the CA was applied to predict the links between all the patient variables during the follow-ups. The AI models’ performance values were rated by using the coefficient of determination (DC) and mean square error (MSE), where the DC concentrates on the accuracy of the model by making sure that the data explained the sufficient goodness of fit, while the MSE minimizes the variables that are less important in the data. The computation of CA is centered on the matrix of rows and columns called dimensions, and it is used to explain the link between the rows and columns for the study variables. Further, the inertia percentage is used to explain the proportion of variation, similar to MCA and PCA. However, before the AI modeling occurred, the data were split into 75% training sets and 25% testing sets. The study secured approval from the research and ethics committee of the Federal Teaching Hospital in Gombe state because it followed the relevant guidelines and regulations of the hospital.

### 2.3. Machine-Learning Models

Machine learning is a technique that uses a computer to perform a specific task, during which the learning algorithm is performed on the basis of the availability of a data set, and it has the potential to address real-world problems and can be used to develop statistical models [19,20,21,22,23,24].

#### 2.3.1. Artificial Neural Network (ANN)

**Figure 1:**
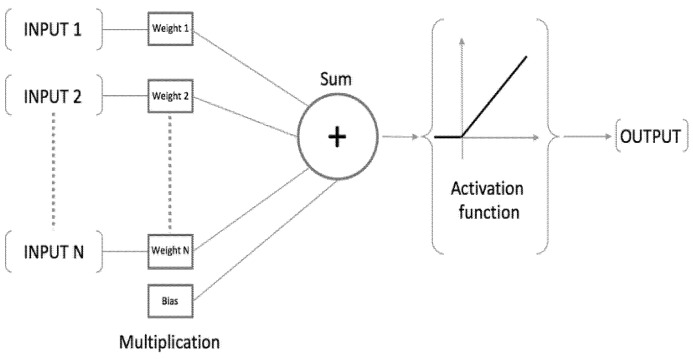
ANN architecture pattern.

The ANN is a biological working structure capable of delivering the best results transformed by inputs without affecting the output operation. It is a class of algorithms with an essential ability to deal with a complicated design data set and can forecast model problems similar to how the human brain can [25,26]. The ANN belongs to a group of algorithms that accepts numeric and structured data to be built on several layers or neurons, such as input, hidden and output, sometimes called multilayer perceptron (MLP). However, the input layer serves as the point of entry for the independent variables into the network system, followed by hidden (intermediate) neurons, which help to connect the computation between the input and the output, while the output layer serves as the final neuron to produce the results [27,28]. The ANN is a function based on the principles of neurons, which serve as processing components assembled in an organized pattern in which each separately connects. As a result, the input is united with the weight and the bias of the network in every single neuron, and the structure levels then act as the primary route for the data to pass through the activation function (see Figure 1). Ref. [29] described MLP by calculating the following function:

$$f:R^{2}\to R,\;y=f\left(x\right)=\emptyset_{2}\left(w_{h0}\emptyset_{1}w_{ih}x+b_{1}\right)+b_{2})$$

where *x* represents the inputs; $\emptyset_{2}$ indicates the function in the output node; $\emptyset_{1}$ represents the activation function at the hidden layer nodes; $w_{ho}$ and $w_{ih}$ define the weight of the matrix linking the output and hidden layers and that of the matrix linking the hidden and the input layers, respectively; and $b_{1}$ and $b_{2}$ represent the bias for the hidden and output layers, respectively.

#### 2.3.2. Support-Vector Machine (SVM)

**Figure 2:**
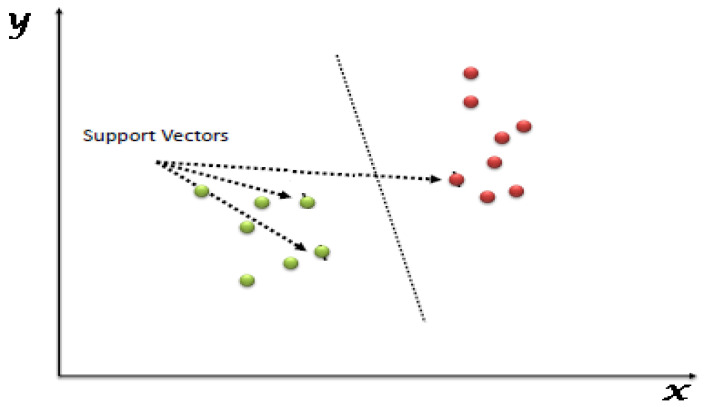
Support-vector machine.

The term SVM in machine learning fits under the category of supervised learning models; it makes use of learning algorithm characteristics to perform analyses for classification and regression [30]. The data can be divided into categories by using the SVM, and the data can then be transformed so that the partition line can be shown as a hyperplane. As a result, the SVM’s operation is focused on classifying data points and then mapping them onto high-dimensional feature space, particularly for data types that are challenging to separate linearly (see Figure 2) [31]. The SVM has more reasonable memory efficiency, capable of handling a clear separation of margin that involves classes. It is more effective in high-dimensional space, especially when the number of dimensions is greater than the number of samples. However, the SVM has major setbacks in dealing with larger values, it is affected by errors, and it performs worse when the traits for each data point surpass the number of samples for the training data and when it has no probabilistic explanation for the classification. Vapnik [17] have stated the ability to conduct learning ideas to achieve the prediction, modeling, regression and categorization of data is the major aim of support-vector machines. The inputs and outputs for the learning process of the modeling are created by $\left(x_{1},y_{i}\right)\;.\;.\;.\;\left(x_{i},y_{i}\right)$. The SVM ensures that it estimates the dependent function of the dependent variable $\left(y\right)$, focusing on the independent variable $\left(x\right)$. Ref. [31] revealed that approximating the function $f\left(x\right)$ is determined by making use of the entire pairs of $\left(x_{1},y_{i}\right)$ alongside the minimum precision $\left(\varepsilon \right)$, and thereafter, the function of Vapnik is named alongside as ε−, the incentive loss function [30,31].

#### 2.3.3. Neuro-Fuzzy (NF)

Neuro-fuzzy is a technique used for resolving uncertainty events, which is used in the uncertainty about human thinking to explain the knowledge through numeric computation on the basis of the imprecise judgment generated by neural networks. NF can use intelligence techniques to handle data processing by using neural networks and fuzzy logic. It is utilized as a concept of partially true and partially false outcomes that can address real-world estimate processes and physical functions, classed as Mamdani, Sugeno and Tsumoto, in which the surgeons produce a larger performance application. The operation of NF is focused on the fuzzy system, which can be manipulated by using neural network principles to train the learning algorithms. The NF is very simple and has acceptable quality, but the rationale is not always exact, because it is sometimes misidentified as a likelihood hypothesis, and it has a well-taught assignment for guiding accuracy and enrollment. Other researchers viewed NF as the process of learning an algorithm from sample data during the modeling process to construct a neural network that can handle specific parameters. Inputting data into fuzzy values for any amendment that requires membership functions (MFs), which are categorized as follows: Gaussian, trapezoidal and triangular sigmoid. They can manage a range of values between 0 and 1. Despite functioning as the membership function, it is designed to link the input performance and the output performance. Therefore, the fuzziness is based on $x_{1}$ and $x_{2}$ as the input and the output f, which will generate the first-order Sugeno fuzziness as follows:

**Theory** **1.** *Assuming $\mu \left(x_{1}\right)$ is $A_{1}$ an $\mu \left(x_{2}\right)$ is $B_{1}$ then $f_{1}=p_{1}x_{1}+q_{2}x_{2}$*.

**Figure 3:**
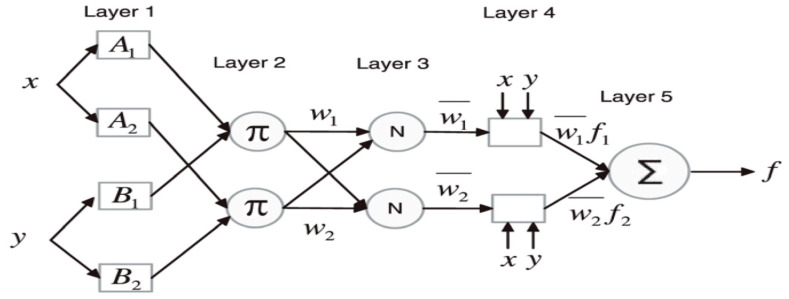
Sketching of neuro-fuzzy application.

**Theory** **2.** *Assuming $\mu \left(x_{1}\right)$ is $A_{2}$ and $\mu \left(x_{2}\right)$ is $B_{2}$ then $f_{2}=p_{2}x_{2}+q_{2}x_{2}$*.

where $A_{1}$, $A_{2}$ and $B_{2}$ explain the constraints and $x_{1}\;\mathrm{and}\;x_{2}$ serve as the membership function while the inputs $p_{1},\;p_{2},\;q_{1},\;q_{2},r_{1}\;\mathrm{and}\;r_{2}$ as the output parameter function, and the planning structure of ANFIS was developed on the basis of five layers of neural network design (see Figure 3).

#### 2.3.4. Multiple Linear Regression (MLR)

**Figure 4:**
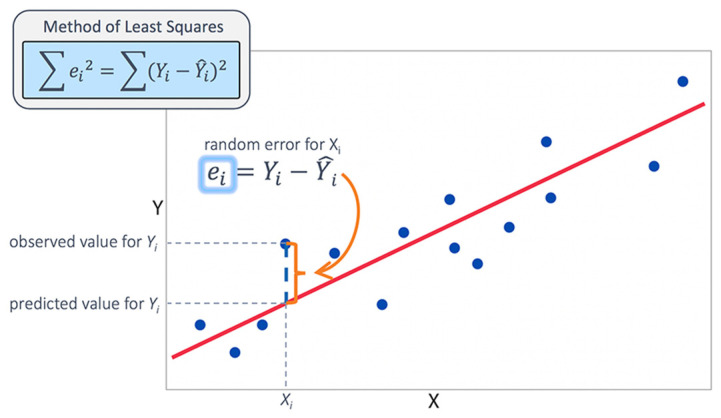
Multiple linear regression.

MLR is a concept that can produce a straight line and simultaneously can estimate the bond between numeric dependent variables and that between two or more independent variables. Granting the researcher access to determining the strength of the relationship between a response variable on various predictor variables is the major operation of the MLR. Aside from the underfitting issues, the MLR is a user-friendly technique that, when used, can produce acceptable results for easier understanding and interpretation within the possible time by using a simple mathematical equation, as seen in Figure 4. MLR is a mathematical approach that can model the relationship between numerous explanatory (independent) factors and a dependent or scalar variable. Linear regression modeling is carried out in two ways: simple linear, which uses y with only an independent variable, and multiple linear, which uses y with more than one predictor variable. Hence, it can be achieved with linear function data. MLR is used to examine the typical conditions of how a mean variable linearly depends on a set of coefficients, while the error term is computed by using a Gaussian distribution.

### 2.4. Correspondence Analysis (CA)

The CA is a dimensional space or dimensional reduction approach that can reduce as much data to the bare-minimum level and then portray it as a cloud. The CA shares similar characteristics of application to PCA, MCA and FA, especially when the number of variables can be fitted in a two-way table. Table 1 features the rows and the columns forming a two-by-three contingency table. CA was defined as a multivariate analysis that deals with the computation of statistics by using exploratory strategies to examine correlations between variables; it uses a similar pattern to that of PCA in terms of finding a relationship between two variables, and both may visually communicate their respective idea in a low-dimensional design. By categorical data in the form of a family tree, remarkable progress has been achieved in statistical applications on the basis of the pattern of association between them. As a consequence of the reciprocal average, centroid position, optimal scaling and homogeneity calculation, it is displayed in the form of a family tree. Similarly, CA is a multidimensional nonlinear scaling or bivariate network analysis that deals with dual scaling, homogeneity analysis, quantification theory, optimal scaling and the method of reciprocal averages. CA allows us to examine how two groups interact in a two- or three-dimensional plot on the basis of using correlations between two or more categories of data. Additionally, CA has a strategy for exploring categorical data using exploratory analysis.

Literature showed that the capability of the CA to analyze research topics in various fields encouraged scholars from different fields to utilize it for analyzing categorical variables. CA has become more popular in ecological research and marketing research because researchers in these areas frequently collect categorical data thanks to the data’s simplicity. It was proved that CA is a multivariate graphical tool for exploring correlations among categorical variables, which is useful in epidemiology to access relationships between variables. The usual purpose of CA is to graphically represent these relative frequencies according to the distance between individual row and column profiles from a contingency table and the distance to the average row and column profile, respectively, so that the relationship among the variables can be visualized in a low-dimensional space. However, CA distance is measured using a chi-square, defined as the metric between row i and row $i^{\prime }\left(i\;\neq \;i^{\prime }\right),$ given by

$$d\left(i,i^{\prime }\right)=\frac{\sum_{j}\left(p_{ij}-p_{i^{\prime }j}\right)}{p_{+j}}$$

where $p_{ij}$ and $p_{i^{\prime }j}$ are the relative frequencies for row i and $i^{\prime }$ in column j and $p_{+j}$ is the marginal relative frequency or mass for i. Therefore, the chi-square distance for two dimensions is defined as $\wedge^{2}=\sum p_{i+}d_{i}$, and

$$d_{i}=\frac{\sum_{j}{\left(p_{ij}-p_{ij}\right)}^{2}}{p_{ij}}$$

denotes the chi-square distance between the row profile and the average row profile.

## 3. Results and Discussion

### 3.1. Results of the AI Models

**Figure 5:**
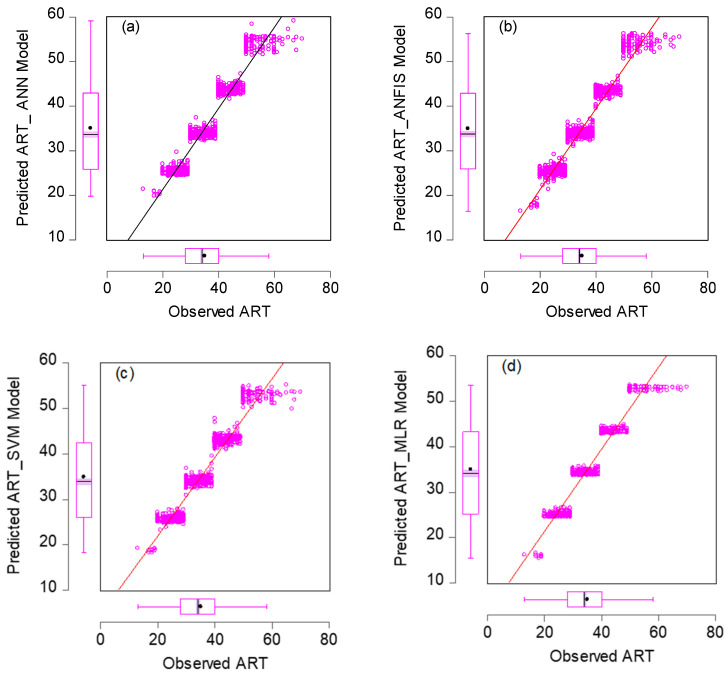
Scatter plots for (**a**) ANN, (**b**) ANFIS, (**c**) SVM and (**d**) MLR models in the training phase.

**Figure 6:**
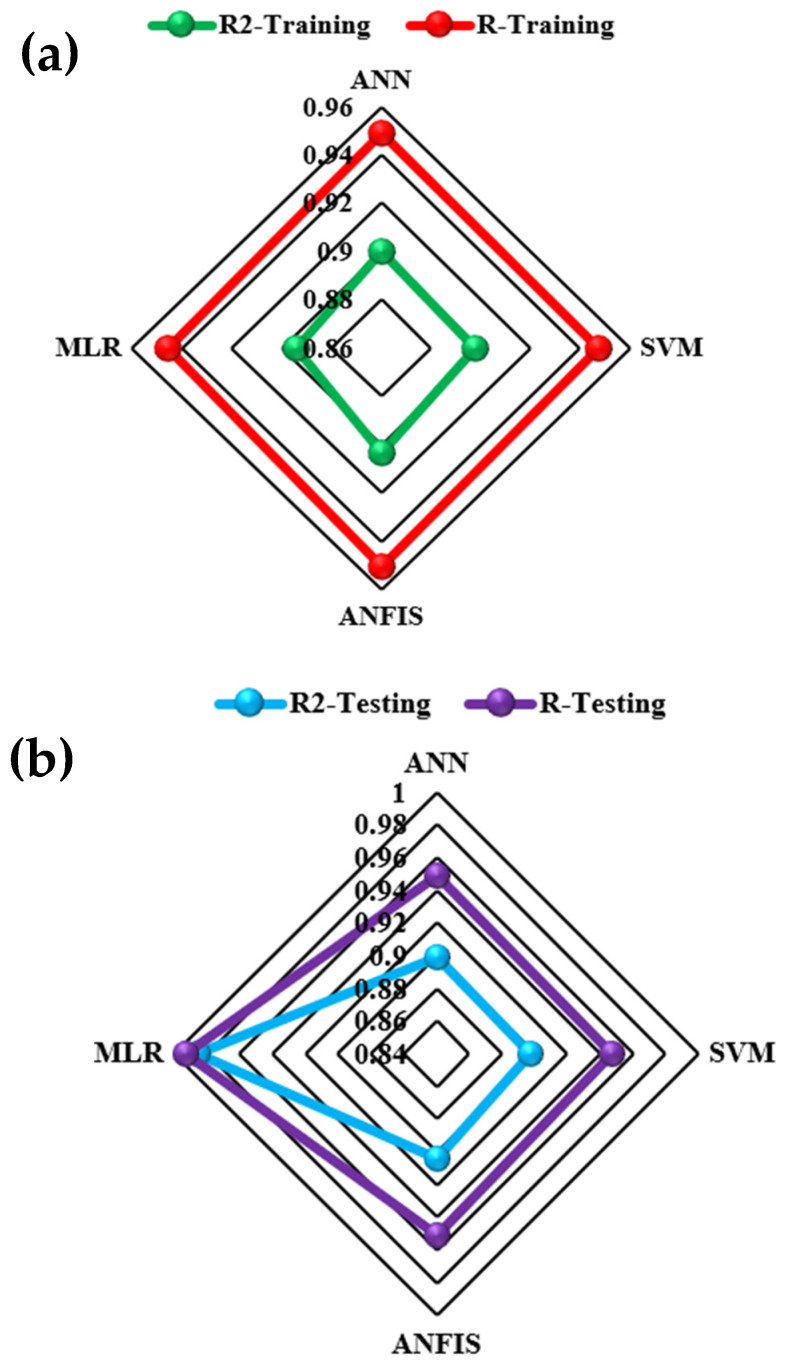
Radar plot for (**a**) training and (**b**) testing performance.

**Figure 7:**
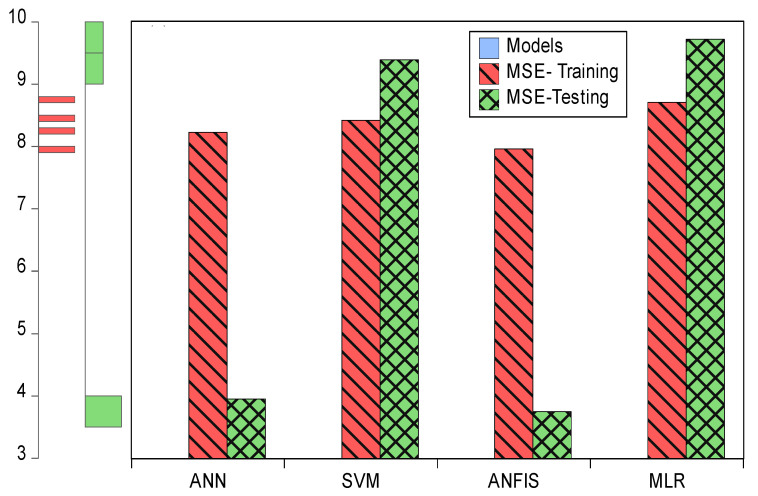
Error plots for MSE in both training and testing.

**Figure 8:**
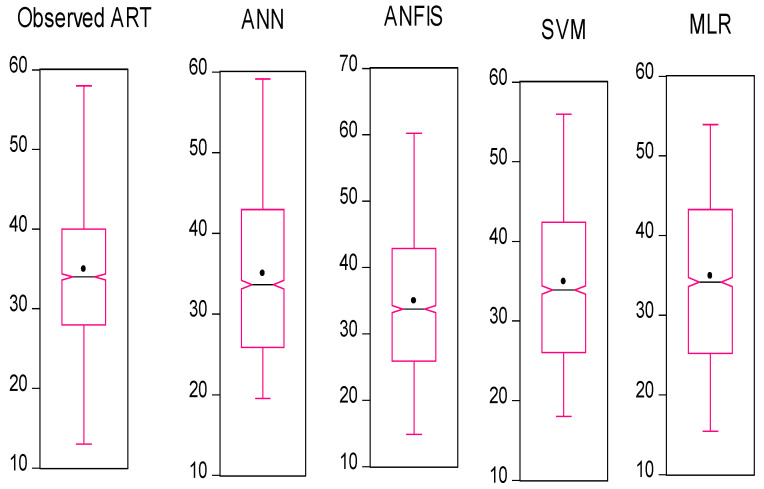
Box plot and whisker plot for all the models.

In this section, we used HIV/AIDS patients receiving ART at the Federal Teaching Hospital in Gombe state to predict the number of years during which patients took ART drugs, by using nonlinear (ANN, SVM and ANFIS) and linear (MLR) AI models, and the CA was applied to examine, during follow-ups, how the patients’ variables contributed to or were associated with ART medication. Criteria evaluation indices, such as coefficient of determination (DC), known as R-square (R^2^) value; mean square error (MSE); percentage of inertia; and dimensions, were used to achieve the results of this analysis. The single models in both training and testing for the ANN, SVM, ANFIS and MLR gave R^2^ values of 0.90–0.89, 0.89–0.89, 0.89, 0.89, 0.90–0.90 and 0.89–0.98 respectively. Additionally, their MSE gave 8.23–3.95, 8.42–9.38, 7.96–3.75 and 8.71–9.72, respectively—as seen in Table 2. According to some researchers presented R^2^ as a tool for measuring the goodness of fit of the model, which is capable of measuring the dependent variable’s projected success from the independent variables, which has a better chance of explaining the proportion of variance by using the regression model. Further, the MSE is the deviation or distance of a value from a hypothetical, unobserved (unexplained) value. Therefore, the ANFIS model outperforms the remaining models, with the lowest error in both the training and testing phases. It has proved to be a unique and promising model capable of handling nonlinear data (see Figure 5, Figure 6, Figure 7 and Figure 8).

### 3.2. Correspondence Analysis Results and Discussion

#### 3.2.1. Descriptive Results for Contingency Table

CA involves the application of a matrix to form a rectangle, which in return gives a contingency table. The rows and columns refer to the marginal frequency count in the contingency table. In this section, all the tables have two types of qualitative variables, such as drugs with marital status in Table 3, drugs with hospital status in Table 4, drugs with a state visit in Table 5 and drugs with age group in Table 6. The study used 2500 patients receiving HIV/AIDS treatment at the Federal Teaching Hospital in Gombe state. Each table has an active margin (marginal row and marginal column) attached to it. In Table 3, drugs are presented in the row, while marital status is presented in the column, revealing that marital status for those that were married (1703) has the highest total number of patients on ART, while divorced (89) has the least total number of patients on ART. The drug ABC-DDI-LPV/r (682) was the most common drug combination that patients used, while thedrug AZT-3TC-EFV (117) was the least commonly taken drug. The hospital status of alive patients (2407), in Table 4, gave a higher number of patients that are doing well, followed by transfer (62) and death (31), in that order, while ABC-DDI-LPV/r remained the most commonly taken drug and AZT-3TC-EFV remained the least commonly taken drug. State visits showed that the mother state, Gombe (1576), had the highest number of patients in attendance, while states (43) outside the North East Zone recorded a lower number of patients in attendance; moreover, ABC-DDI-LPV/r remained the leading drug taken, with AZT-3TC-EFV as the least common drug (see Table 5). Finally, Table 6 shows that the age group 30 to 39 (956) was the group that was most commonly on medication, while the age group 10 to 19 (21) was the group least commonly on medication. ABC-DDI-LPV/r maintained its lead as the highest prescribed drug, and AZT-3TC-EFV was the least prescribed drug.

#### 3.2.2. Relationships among Row Dimensions

In CA, dimensions represent the rows (m) and columns (n) in any given set of data computed using the chi-square table of the matrix. The rows and column tell us whether there is a larger or smaller difference within or among the data set. In each dimension, the variables that have a smaller difference in any row or column have a better relationship or link with the variables, while the variables that have a larger difference in any row or column do not have a good relationship. The *contingency table* (cross-tabulation table), which was developed by a well-known researcher to characterize the measures of divergence from complete independence between the row and column structures based on a rectangle, where the total number of dimensions determines the number of rows or columns present. In this section, we used the row (a combination of ART medications) to study the variable that is closer to zero. Each patient variable in the row that is closer to zero is considered as the variable that meaningfully contributed to the combination of drugs. Table 7 represents the effect of ART medication on married couples, revealing in row 1 that AZT-3TC-NPV (0.001), TDF-3TC-EFV (0.000), TDF-3TC-LPV/r (0.001) and TDF-FTC-NPV (0.000) contributed more to treatment in married couples compared with the remaining drugs, while ABC-DDI-LPV/r (0.003), AZT-3TC-EFV (0.005), TDF-3TC-ATV/r (0.001) and TDF-3TC-EFV (0.005) in row II more meaningfully contributed to treatment for the married couples than did the remaining drugs.

Subsequently, Table 8 explains the relationships between ART drugs for the patients’ hospital status, indicating that row 1 showed that AZT-3TC-ATV/r (0.001), AZT-3TC-LPV/r (0.001), TDF-3TC-EFV (0.001), TDF-3TC-NPV (0.000), TDF-FTC-ATV/r (0.007) and TDF-FTC-NPV (0.000) contributed more to treatment according to hospital status compared with the remaining drugs, while in row II, AZT-3TC-EFV (0.007), AZT-3TC-NPV (0.004) and TDF-3TC-ATV/r (0.005) contributed more to treatment according to hospital status compared with the remaining drugs. The relationship between drugs according to visits to the hospital from various locations showed that ABC-DDI-LPV/r (0.007), AZT-3TC-EFV (0.000), AZT-3TC-LPV/r (0.002), and TDF-FTC-ATV/r (0.000) contributed more to treatment according to state visits, in row I, while in row II, ABC-DDI-LPV/r (0.000), AZT-3TC-NPV (0.004), TDF-FTC-EFV (0.006) and TDF-FTC-LPV/r (0.001) contributed more to treatment according to state visits compared with the remaining drugs (see Table 9).

**Figure 9:**
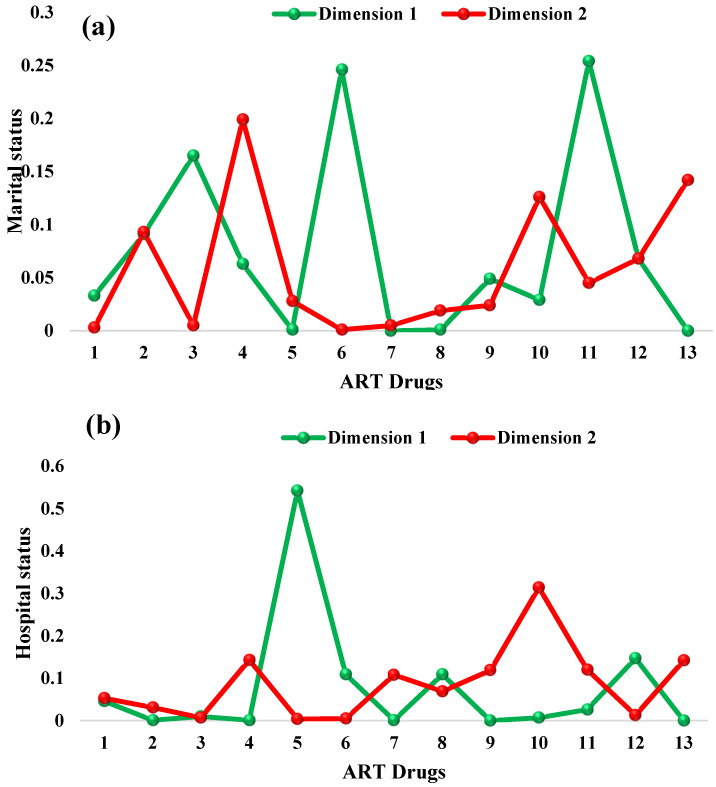

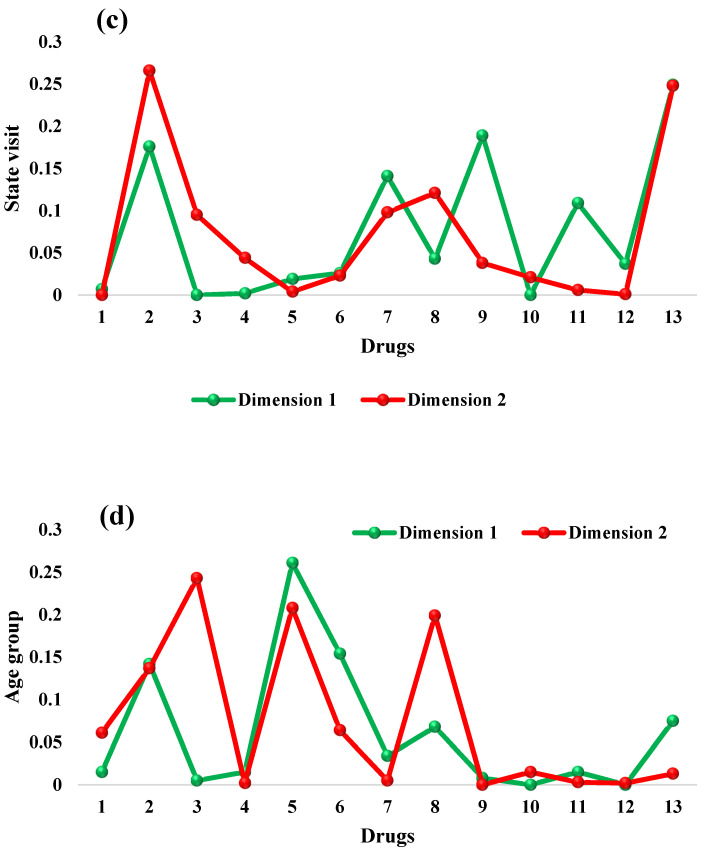
Point contribution to inertia for the row dimensions (ART drugs), measured against (**a**) marital status, (**b**) hospital status, (**c**) state visit and (**d**) age group.

Lastly, the influence of the drugs on patients’ age groups, shown in Table 10, indicated that AZT-3TC-EFV (0.005), TDF-3TC-NPV (0.008), TDF-FTC-ATV/r (0.000) and TDF-FTC-LPV/r (0.000), in row I, contributed more to treatment according to age group, while AZT-3TC-LPV/r (0.002), TDF-3TC-EFV (0.005), TDF-3TC-NPV (0.000), TDF-FTC-EFV (0.003) and TDF-FTC-LPV/r (0.002), in row II, contributed more to treatment according to age group compared with the remaining drugs. Figure 9 shows the contribution of inertia to the row dimensions (ART drug) as measured against marital status, hospital status, state visit and age group.

#### 3.2.3. Relationships among Column Dimensions

**Figure 10:**
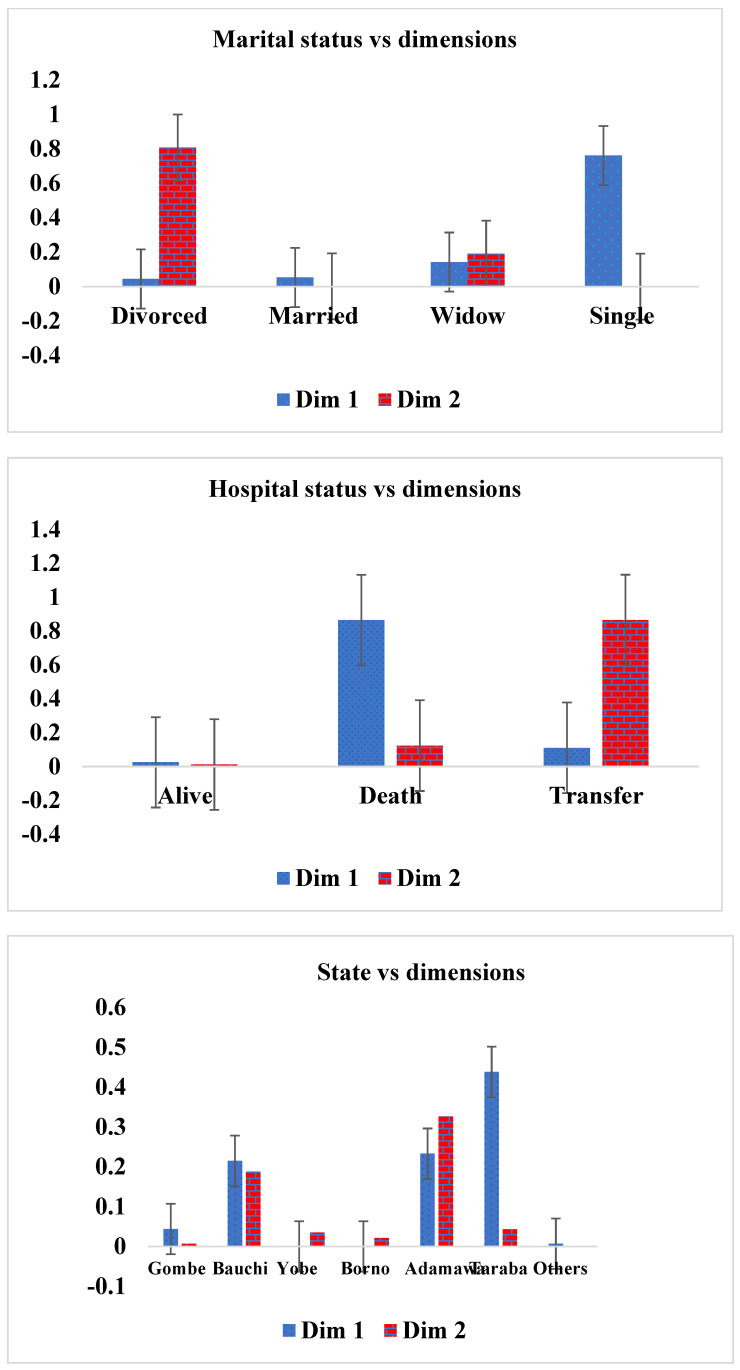

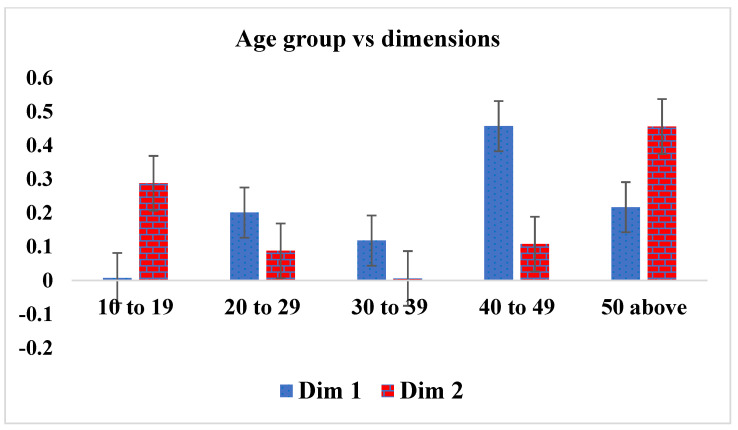
Contribution to inertia for the column dimensions (ART drugs) against marital status, hospital status, state visit and age group.

In this section, we use the column (marital status, hospital status, state visit and age group) results to examine those variables that are closer to zero. Each patient variable in the column that is closer to zero is considered as the variable that meaningfully contributed to the study’s demographic variables. Therefore, the relationship between marital status and a patient placed on an ART drug showed that divorced (0.044) and married (0.053) patients are doing well on ART medication compared with the remaining marital statuses, in column 1, while in column II, married (0.001) and single (0.000) patients are doing better on treatment than the other marital statuses (see Table 11) are. Consequently, as shown in Table 12, alive patients (0.025) are doing better on ART medication, in column 1 and column II (0.025 and 0.012), compared with the remaining hospital statuses. Table 13 showed that patients visiting the hospital from Yobe (0.000), Borno (0.000) and other areas outside the northeast zone (0.007), in column I, have a better relationship with treatment, while patients visiting from Gombe (0.007) state and other states (0.000), in column II, contributed more to drug treatment compared with those from the remaining states who were visiting the hospital. Finally, age groups 10–19 (0.007) and 30–39 (0.006), as shown in column I and column II of Table 14, are doing better on treatment compared with the other age groups. Figure 10 represents the contribution of inertia to the column dimensions (marital status, hospital status, state visit and age group), for patients placed on an ART drug.

#### 3.2.4. Contingency Table Results for CA, Chi-Square and Percentage of Explained Variation

**Figure 11:**
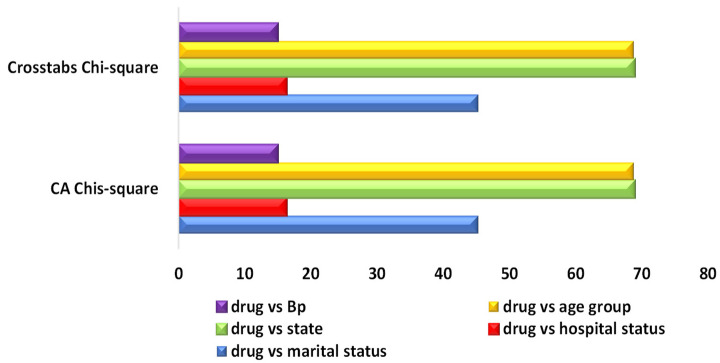
CA and crosstab results analysis.

The results for the CA and chi-square test, as shown in Table 15, were similar to the results in several other studies in the literature (see Figure 11). Comparisons of the results from both the CA and the chi-square test showed the following: drugs and marital status gave ($\chi^{2}$ = 45.252, *p* = 0.139 and R^2^ = 93.7%), drugs and hospital status gave ($\chi^{2}$ = 16.334, *p* = 0.876, and R^2^ = 70.6%), drugs and state visit gave ($\chi^{2}$ = 68.886, *p* = 0.582, and R^2^ = 58.9%) and drugs and age group gave ($\chi^{2}$ = 68.733, *p* = 0.026, and R^2^ = 79.4%). This showed only that the relationship drugs and age group was significant (*p* = 0.026), and the remaining variables (marital status, hospital status and state visit) were not significant. Nonetheless, drugs and marital status (93.7%) explained the highest total proportion of variation compared with the remaining variables, as indicated in Table 15. It was confirmed CA as a statistical approach that uses the same standards as the chi-square approach, aiming at measuring the weight and distance between the exhibited points that appear on the biplot.

## 4. Conclusions

A combination of artificial intelligence models and correspondence analysis techniques was employed in this study to predict and examine HIV/AIDS-treated patients at the Federal Teaching Hospital in Gombe state. The single nonlinear model and linear models (ANN, ANFIS, SVM and MLR), including CA, were measured by using evaluation index criteria terms such as R^2^, MSEs, percentages of inertia, dimensions, active margins, *p*-values and chi-square tests. The comparison results for the study showed that ANFIS R^2^ (0.903 and 0.904) with MSE (7.961 and 3.751) outperformed the remaining models in both training and testing. This revealed that patients’ lives become safer and healthier as their time on ART medicine increases. The descriptive results showed that ABC-DDI-LPV/r (682) was the most prescribed drug for the patients. Marital status for those that were married (1703) recorded a high number of patients in attendance; hospital status showed alive patients (2407) on treatment were doing well in that only a few deaths were recorded while patients received ART treatment; a state visit showed that Gombe (1576) has the highest number of patients in attendance; and the 30 to 39 age group (956 patients) was the group most committed to undergoing treatment.

The results from the dimensions showed that drugs AZT-3TC-NPV (0.001), TDF-3TC-EFV (0.000), TDF-3TC-LPV/r (0.001) and TDF-FTC-NPV (0.000) contributed more according to marital status, in row I, compared with the remaining drugs, while in row II, drugs ABC-DDI-LPV/r (0.003), AZT-3TC-EFV (0.005) TDF-3TC-ATV/r (0.001) and TDF-3TC-EFV (0.005) contributed more to marital status than the other drugs did. Subsequently, in row I, AZT-3TC-ATV/r (0.001), AZT-3TC-LPV/r (0.001), TDF-3TC-EFV (0.001), TDF-3TC-NPV (0.000), TDF-FTC-ATV/r (0.007) and TDF-FTC-NPV (0.000) contributed more according to hospital status compared with the other drugs, while AZT-3TC-EFV (0.007), AZT-3TC-NPV (0.004) and TDF-3TC-ATV/r (0.005) contributed more according to hospital status, in row II, compared with the remaining drugs.

Next, ABC-DDI-LPV/r (0.007), AZT-3TC-EFV (0.000), AZT-3TC-LPV/r (0.002) and TDF-FTC-ATV/r (0.000) contributed more according to various state visits to the hospital, in row I, compared with the remaining drugs, while ABC-DDI-LPV/r (0.000), AZT-3TC-NPV (0.004), TDF-FTC-EFV (0.006) and TDF-FTC-LPV/r (0.001) contributed more according to state visits, in row II. Finally, AZT-3TC-EFV (0.005), TDF-3TC-NPV (0.008), TDF-FTC-ATV/r (0.000) and TDF-FTC-LPV/r (0.000) contributed more according to age groups, in row I, while in row II, AZT-3TC-LPV/r (0.002), TDF-3TC-EFV (0.005), TDF-3TC-NPV (0.000), TDF-FTC-EFV (0.003) and TDF-FTC-LPV/r (0.002) contributed more according to age groups compared with the remaining drugs.

However, the dimension results showed that divorced (0.004) and married (0.053) patients were doing better on drugs than the remaining marital statuses, in column I, while in column II, married (0.001) and single (0.000) patients were doing better on the drug compared with the remaining marital statuses. Alive patients (0.025), in column I and column II (0.012), were doing better on drugs than the remaining hospital statuses were. State visit data showed that Yobe state (0.000), Borno state (0.000) and other states (0.007) were doing better on the drug, in column I, compared with the remaining states, while Gombe state (0.007) and other states (0.000), in column II, were doing well on the drug compared with the remaining states. The age-group category revealed that age group 10–19 (0.007), in column I, and age group 30–39 (0.006), in column II, were doing better on the drug than the remaining age groups. The CA and the chi-square test yielded similar results, revealing that only the relationship between drug and age group was significant ($\chi^{2}$ = 68.733, *p* = 0.026, and R^2^ = 79.4%), but in terms of performance, the relationship between drugs and marital status (93.7%) explained a higher percentage of variation compared with what the remaining variables could. A combination of different AI models and multivariate exploratory analysis techniques are required in future studies to evaluate epidemiological data other than HIV data.

## Figures and Tables

**Table 1:** Cross-tabulation of 2 × 3 contingency table (gender vs hospital status).

Gender/Hospital		Alive	Death	Transfer
Male	Count	519	9	7
	Expected Count	517.5	6.4	11.1
	% within gender	97.0%	1.7%	1.3%
	% within hospital status	35.8%	50.0%	22.6%
Female	Count	932	9	24
	Expected Count	933.5	11.6	19.9
	% within gender	96.6%	0.9%	2.5%
	% within hospital status	64.2%	50.0%	77.4%

**Table 2:** Training and testing performance of single models.

Training					Testing			
Model	R^2^	R	MSE	RMSE	R^2^	R	MSE	RMSE
ANN	0.90007	0.94872	8.228	2.868	0.899249	0.948287	3.949	1.987
SVM	0.89777	0.94751	8.417	2.901	0.897361	0.947292	9.387	3.064
ANFIS	0.90332	0.95043	7.961	2.821	0.904295	0.950944	3.751	1.936
MLR	0.89425	0.94565	8.707	2.951	0.985961	0.992956	9.717	3.117

**Table 3:** Drug vs marital status.

ART Drugs	Divorced	Married	Widow	Single	Active Margin
ABC-DDI-LPV/r	23	464	88	107	682
AZT-3TC-ATV/r	2	72	15	11	100
AZT-3TC-EFV	3	74	12	28	117
AZT-3TC-LPV/r	9	101	14	18	142
AZT-3TC-NPV	8	137	19	33	197
TDF-3TC-ATV/r	8	153	27	21	209
TDF-3TC-EFV	9	121	21	31	182
TDF-3TC-LPV/r	11	114	20	30	175
TDF-3TC-NPV	5	89	11	26	131
TDF-FTC-ATV/r	2	107	19	30	158
TDF-FTC-EFV	5	89	9	33	136
TDF-FTC-LPV/r	2	91	15	28	136
TDF-FTC-NVP	2	91	19	23	135
Active Margin	89	1703	289	419	2500

**Table 4:** Drug vs hospital status.

Drug	Alive	Death	Transfer	Active Margin
ABC-DDI-LPV/r	661	7	14	682
AZT-3TC-ATV/r	96	1	3	100
AZT-3TC-EFV	113	1	3	117
AZT-3TC-LPV/r	138	2	2	142
AZT-3TC-NPV	184	6	7	197
TDF-3TC-ATV/r	204	1	4	209
TDF-3TC-EFV	174	2	6	182
TDF-3TC-LPV/r	167	4	4	175
TDF-3TC-NPV	127	2	2	131
TDF-FTC-ATV/r	151	1	6	158
TDF-FTC-EFV	129	2	5	136
TDF-FTC-LPV/r	133	0	3	136
TDF-FTC-NVP	130	2	3	135
Active Margin	2407	31	62	2500

**Table 5:** Drug vs state visit.

Drug	Gombe	Bauchi	Yobe	Borno	Adamawa	Taraba	Others	Active Margin
ABC-DDI-LPV/r	423	98	48	35	49	15	14	682
AZT-3TC-ATV/r	58	9	10	7	8	6	2	100
AZT-3TC-EFV	71	13	12	7	9	2	3	117
AZT-3TC-LPV/r	87	17	13	10	10	2	3	142
AZT-3TC-NPV	128	28	16	11	7	4	3	197
TDF-3TC-ATV/r	132	30	13	8	13	5	8	209
TDF-3TC-EFV	115	32	8	9	14	2	2	182
TDF-3TC-LPV/r	110	21	17	7	16	3	1	175
TDF-3TC-NPV	75	27	10	9	8	1	1	131
TDF-FTC-ATV/r	95	22	11	12	12	5	1	158
TDF-FTC-EFV	93	15	11	6	5	4	2	136
TDF-FTC-LPV/r	92	16	10	5	11	1	1	136
TDF-FTC-NVP	97	17	5	7	2	5	2	135
Active Margin	1576	345	184	133	164	55	43	2500

**Table 6:** Drug vs age group.

Drug	10 to 19	20 to 29	30 to 39	40 to 49	50 and Above	Active Margin
ABC-DDI-LPV/r	5	214	283	139	41	682
AZT-3TC-ATV/r	1	22	36	34	7	100
AZT-3TC-EFV	3	34	48	26	6	117
AZT-3TC-LPV/r	1	43	50	37	11	142
AZT-3TC-NPV	1	56	59	54	27	197
TDF-3TC-ATV/r	0	72	88	34	15	209
TDF-3TC-EFV	1	48	71	41	21	182
TDF-3TC-LPV/r	0	63	67	29	16	175
TDF-3TC-NPV	1	34	47	36	13	131
TDF-FTC-ATV/r	2	48	60	36	12	158
TDF-FTC-EFV	3	51	45	25	12	136
TDF-FTC-LPV/r	1	42	51	32	10	136
TDF-FTC-NVP	2	33	51	35	14	135
Active Margin	21	760	956	558	205	2500

**Table 7:** Row dimensions for marital status, for patients placed on an ART drug.

ART Drugs	Dimension 1	Dimension 2
ABC-DDI-LPV/r	0.033	0.003
AZT-3TC-ATV/r	0.091	0.093
AZT-3TC-EFV	0.165	0.005
AZT-3TC-LPV/r	0.063	0.199
AZT-3TC-NPV	0.001	0.028
TDF-3TC-ATV/r	0.246	0.001
TDF-3TC-EFV	0.000	0.005
TDF-3TC-LPV/r	0.001	0.019
TDF-3TC-NPV	0.049	0.024
TDF-FTC-ATV/r	0.029	0.126
TDF-FTC-EFV	0.254	0.045
TDF-FTC-LPV/r	0.068	0.068
TDF-FTC-NVP	0.000	0.142

**Table 8:** Row dimensions for hospital status, for patients placed on an ART drug.

ART Drugs	Dimension 1	Dimension 2
ABC-DDI-LPV/r	0.046	0.053
AZT-3TC-ATV/r	0.001	0.031
AZT-3TC-EFV	0.010	0.007
AZT-3TC-LPV/r	0.001	0.143
AZT-3TC-NPV	0.542	0.004
TDF-3TC-ATV/r	0.109	0.005
TDF-3TC-EFV	0.001	0.108
TDF-3TC-LPV/r	0.109	0.069
TDF-3TC-NPV	0.000	0.119
TDF-FTC-ATV/r	0.007	0.314
TDF-FTC-EFV	0.026	0.120
TDF-FTC-LPV/r	0.147	0.013
TDF-FTC-NVP	0.000	0.142

**Table 9:** Row dimensions for state visits, for patients placed on an ART drug.

Drug	Dimension 1	Dimension 2
ABC-DDI-LPV/r	0.007	0.000
AZT-3TC-ATV/r	0.176	0.266
AZT-3TC-EFV	0.000	0.095
AZT-3TC-LPV/r	0.002	0.044
AZT-3TC-NPV	0.019	0.004
TDF-3TC-ATV/r	0.026	0.023
TDF-3TC-EFV	0.141	0.098
TDF-3TC-LPV/r	0.043	0.121
TDF-3TC-NPV	0.189	0.038
TDF-FTC-ATV/r	0.000	0.021
TDF-FTC-EFV	0.109	0.006
TDF-FTC-LPV/r	0.037	0.001
TDF-FTC-NVP	0.249	0.248

**Table 10:** Row dimensions for age group, for patients placed on an ART drug.

Drug	Dimension 1	Dimension 2
ABC-DDI-LPV/r	0.015	0.061
AZT-3TC-ATV/r	0.142	0.137
AZT-3TC-EFV	0.005	0.243
AZT-3TC-LPV/r	0.015	0.002
AZT-3TC-NPV	0.261	0.208
TDF-3TC-ATV/r	0.154	0.064
TDF-3TC-EFV	0.034	0.005
TDF-3TC-LPV/r	0.068	0.199
TDF-3TC-NPV	0.008	0.000
TDF-FTC-ATV/r	0.000	0.015
TDF-FTC-EFV	0.015	0.003
TDF-FTC-LPV/r	0.000	0.002
TDF-FTC-NVP	0.075	0.013

**Table 11:** Column dimensions for marital status, for patients placed on an ART drug.

Marital Status	Dimension 1	Dimension 2
Divorced	0.044	0.808
Married	0.053	0.001
Widow	0.142	0.191
Single	0.761	0.000

**Table 12:** Column dimensions for hospital status, for patients placed on an ART drug.

Hospital Status	Dimension 1	Dimension 2
Alive	0.025	0.012
Death	0.865	0.123
Transfer	0.110	0.865

**Table 13:** Column dimensions for state visit, for patients placed on an ART drug.

State	Dimension 1	Dimension 2
Gombe	0.044	0.007
Bauchi	0.215	0.188
Yobe	0.000	0.035
Borno	0.000	0.021
Adamawa	0.233	0.327
Taraba	0.438	0.043
Others	0.007	0.000

**Table 14:** Column dimensions for age group, for patients placed on an ART drug.

Age Group	Dimension 1	Dimension 2
10 to 19	0.007	0.288
20 to 29	0.201	0.088
30 to 39	0.118	0.006
40 to 49	0.457	0.108
50 and above	0.217	0.456

**Table 15:** CA, chi-square and percentage of variation results.

	CA Result		Chi-Square Result		
	Chis-square	*p*-value	Chi-square	*p*-value	R-square
Drugs vs marital status	45.252	0.139	45.252	0.139	93.7%
Drugs vs hospital status	16.334	0.876	16.334	0.876	70.6%
Drugs vs state	68.886	0.582	68.886	0.582	58.9%
Drug vs age group	68.733	0.026	68.733	0.026	79.4%

## Data Availability

Not applicable.
